# Whole-body vibration training improves muscle mass and strength in older adults through intra- and extra-muscular pathways

**DOI:** 10.3389/fcell.2025.1643478

**Published:** 2025-10-24

**Authors:** Xiangyang Tian, Shaoting Fu, Jing He, Rui Ma, Rengfei Shi

**Affiliations:** ^1^ School of Exercise and Health, Shanghai University of Sport, Shanghai, China; ^2^ The Key Laboratory of Exercise and Health Sciences of Ministry of Education, Shanghai University of Sport, Shanghai, China; ^3^ College of Physical Education, Shanghai Normal University, Shanghai, China

**Keywords:** whole-body vibration training, sarcopenia, neuromuscular junction transmission, muscle fiber capillarization, protein synthesis and protein degradation, myogenesis

## Abstract

Sarcopenia, a geriatric syndrome characterized by the age-related loss of muscle mass and function, is increasingly recognized to be a dynamic process and exists bidirectional transitions with both possible worsening or improving transitions. With the rapid growth of aging population, developing effective interventions to delay or prevent the progression of sarcopenia is important and urgent. Recently, growing evidence demonstrated that whole body vibration training (WBVT) could significantly improve muscle mass and/or muscle strength in older adults, and may be a promising approach for elderly adults to treat sarcopenia, but there still exists inconsistent results. To clarify the moderating variables affecting the effectiveness of WBVT on muscle mass and strength, we conducted a comprehensively search of electronic database (PubMed, Web of Science and Embase) and performed subgroup analysis depending on the characteristics of subjects (sarcopenia vs. non-sarcopenia), frequency and amplitude (low frequency low amplitude, low frequency high amplitude, high frequency low frequency and high frequency high amplitude) and body position. We found that WBVT significantly enhanced muscle strength in older adults with or without sarcopenia, and the improvements on muscle strength were greatest after WBVT intervention with high frequency high amplitude, compared with WBVT with low frequency low amplitude, low frequency high amplitude, high frequency low frequency; WBVT only increased muscle mass in non-sarcopenic individuals, body position may be an important factor influencing the effectiveness of WBVT, compared with static body position, dynamic body position during WBVT has beneficial effects on muscle mass in older adults. Furthermore, skeletal muscle contraction is under the control of motor neuron and consumes a large amount of oxygen. The factors from intra-muscular including the protein synthesis and degradation and the proliferation and differentiation of satellite cells, to extra-muscular such as microcirculation and motor neuron control are all crucial for the maintenance of muscle mass and strength, therefore, we reviewed the regulatory effects of WBVT on these indicators, which would deepen our understandings about the mechanisms about the effects of WBVT on muscle mass and strength.

## 1 Introduction

Skeletal muscle tissue is not only responsible for force generation during exercise, but also acts as the largest endocrine organ participating in the regulation of multiple tissues or organs. During aging, muscle mass and function occurs progressive decrease, which was also termed as sarcopenia. Growing evidence demonstrated that sarcopenia not only increased the risks of falls and fracture (Yeung et al., 2019) and functional disability (Beaudart et al., 2023; Zhou et al., 2024), but also closely associated with the occurrence and development of multiple metabolic disease, such as type 2 diabetes, non-alcoholic fatty liver disease and cardiovascular diseases (He et al., 2024; Lu et al., 2024). As global ageing accelerates, the prevalence of sarcopenia raises parallelly, it is estimated that the number of elderly people with sarcopenia will increase up to 200 million by 2060 (Alves et al., 2023). The European Working Group on Sarcopenia in Older People (EWGSOP) initially recommended using reduced muscle mass combined with low muscle function (strength or performance) as the clinical diagnosis of sarcopenia (Cruz-Jentoft et al., 2010). However, to enhance the awareness and care for sarcopenia, EWGSOP updated the definition in 2018 (EWGSOP2) which emphasized low muscle strength as a key characteristic of sarcopenia, and pointed out that sarcopenia is probable when low muscle strength occurred, and diagnosed by the presence of low muscle quantity or quality. When low muscle strength, low muscle quantity/quality and low physical performance are all detected, it will be considered as severe sarcopenia (Cruz-Jentoft et al., 2019). At the same time, considering the differences in anthropometric and lifestyle between Asian and Western contemporaries, the Asian Working Group for Sarcopenia (AWGS) also updated its expert consensus in 2019 (AWGS2019), and introduced the concept of possible sarcopenia (defined by either low muscle strength or reduced physical performance only) to identify early individuals who are at risk and perform timely intervention (Chen et al., 2020).

It has been increasingly recognized that the development of sarcopenia is a dynamic process with both possible worsening or improving transitions. More and more evidence showed that the development of sarcopenia could occur in a relatively short period, despite it is commonly perceived as a progressive process. A longitudinal cohort study in community-dwelling older adults found that 13.5% of males and 11.7% of females who were initially diagnosed as non-sarcopenic individuals developed sarcopenia during a 2-year follow-up period (Choe et al., 2022). Another study conducted on dialysis patients also demonstrated that 24.9% of individuals transitioned from non-sarcopenia to sarcopenia over a 1-year follow-up period (Yang Y. et al., 2023). Additionally, once the elderly people developed sarcopenia, they would have a lower likelihood of recovering to non-sarcopenia than those with possible or probable sarcopenia. The longitudinal evidence from the China Health and Retirement Longitudinal Study (CHARLS) showed that 24.5% of individuals with possible sarcopenia returned to non-sarcopenia during a 3.29-year follow up period, while only 14.3% of patients with sarcopenia recovered to non-sarcopenia (Luo et al., 2024). A population-based study estimated the transition probabilities across sarcopenia stages through continuous-time multistage Markov model, and found that subjects with probable sarcopenia had a 10.7% chance of reverting to no sarcopenia, while the probability of reverting to no sarcopenia among participants with sarcopenia was only 3.4%, and with a 70.9% chance of dying after 10 years (Trevisan et al., 2021), which was similar to the results of Sun et al. that a 2-way dynamic process with both progression and reversion across sarcopenia states, and the probability of reversing to no sarcopenia was greater among possible sarcopenic older adults, compared with sarcopenic participants (Sun et al., 2023). Therefore, implementing effective interventions timely is of great importance to prevent and reduce the burdens of sarcopenia.

However, there is no particularly effective pharmacological treatment for sarcopenia at present (Cruz-Jentoft and Sayer, 2019). The World Health Organization recommends older people to perform moderate-intensity aerobic exercise for 150 min per week or undertake high-intensity aerobic exercise for 75 min per week, combined with resistance exercise 2–3 times per week to prevent chronic or debilitating conditions and/or treat disease. Evidence from randomized controlled trails (RCTs) and meta-analysis demonstrated that multiple types of exercise, such as resistance training, mixed aerobic, strength and balance training, had positive effects on muscle mass, strength and physical performance in older adults with sarcopenia or low muscle function (Lu et al., 2021; Monti et al., 2023; Shen et al., 2023), so it was recommended as a non-pharmacological intervention for sarcopenia. However, most elderly people are unwilling or unable to perform these traditional exercise regimens and have a lower compliance. Whole body vibration training (WBVT) is an emerging training method that uses vibration platform to produce mechanical oscillation stimulus with different vibration frequencies and amplitudes in different positions and induce muscle contraction. It has been widely used in rehabilitation following neurological disease such as stroke (Yang X. et al., 2023; Xu et al., 2024), Parkinson’s disease (Chang et al., 2022) and cerebral palsy (Steinberg et al., 2023). Recently, mounting evidence indicated that WBVT significantly improved muscle mass, strength and physical performance in older adults (Chang et al., 2018; Lu et al., 2022), with higher compliance (reached up to 93%) and no adverse effects, especially in participants who are not able to perform standard exercises (Rogan et al., 2015; Lai et al., 2018; Wu et al., 2020; Tan et al., 2023; Zhuang et al., 2025), so it may be an alternative physical training method to prevent or delay the progression of sarcopenia. But there are conflicting results, and the underlying mechanisms has not been clarified yet. In this review, we firstly clarified the influence of the moderating variables on the effectiveness of WBVT by performed subgroup analysis depending on the characteristics of subjects (sarcopenia vs. non-sarcopenia), frequency and amplitude (low frequency low amplitude, low frequency high amplitude, high frequency low frequency and high frequency high amplitude) and body position, then summarized the effects of WBVT on indicators crucial for muscle mass and strength, which is not only beneficial for community physicians to develop appropriate WBVT programme, but also deepens our understandings about the mechanisms about the effects of WBVT on muscle mass and strength.

## 2 The effects of whole-body vibration training on muscle mass and strength in older adults

A systemic review has reported that vibration therapy has benefits on muscle strength and physical performance in older adults with sarcopenia, but not muscle mass (Wu et al., 2020). As described in Introduction, sarcopenia is a dynamic process with a lower likelihood of recovering to non-sarcopenia, therefore increasing muscle mass and strength in non-sarcopenic older adults may be an effective way to prevent the development. However, it is unknown whether the effectiveness of WBVT on non-sarcopenic older adults was similar or superior to sarcopenic elderly adults, so we conducted a systematic review to compare the effects of WBVT on muscle mass and strength in individuals with or without sarcopenia.

### 2.1 Search strategy

We conducted a comprehensive search of electronic databases (PubMed, Web of Science and Embase), with no limitation of publication year. The search terms used were as follows: (whole-body vibration intervention OR vibration OR vibration therapy OR vibrating OR whole body vibration OR whole-body vibration OR whole-body vibration training OR vibration training OR whole-body vibrating therapy) AND (old adults OR older people OR older adults OR elderly people OR elderly OR aging adults OR advanced old age) AND (muscle mass OR muscle size OR muscle loss OR muscle wasting OR muscular atrophy OR muscle weakness OR sarcopenia OR sarcopenic OR muscle strength OR handgrip strength) NOT (Chronic Obstructive Pulmonary Diseases OR COPD OR Tumor OR Cancer OR Malignancy OR Kidney Diseases OR kidney disease OR stroke OR stroke patients OR cerebral palsy OR multiple sclerosis). The detailed search strategy was listed Supplementary Material File 1.

### 2.2 Eligibility criteria

The inclusion criteria were as follows: (1) age >60 years; (2) without diseases or conditions of COPD, cancer, kidney disease, stroke and diabetes; (3) the study has a WBVT alone group and control group that received a no-exercise intervention; (4) the outcomes included muscle mass and/or muscle strength. Studies were excluded if they failed to meet the inclusion criteria and/or (1) not full-text article; (2) not English; (3) meeting abstracts; (4) animal studies and (5) reviews.

### 2.3 Study selection

**FIGURE 1:**
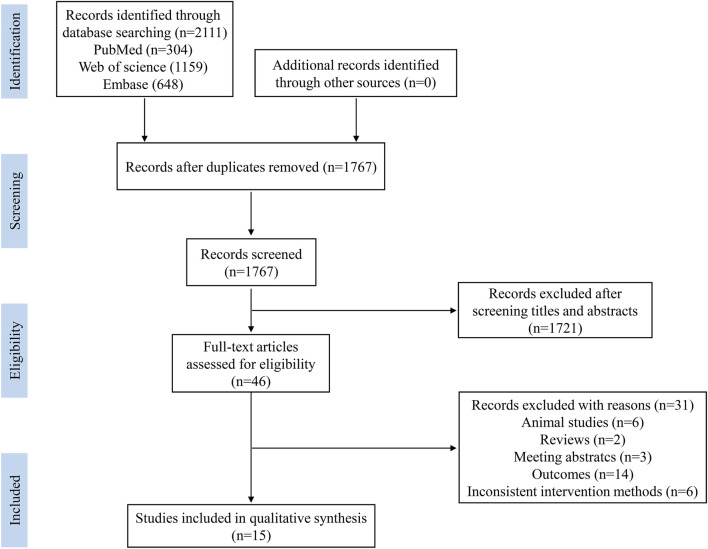
The flowchart of study screening and selection process.

To avoid missing relevant studies, the retrieved literature were saved in reference manager software (EndNote X21, Thomson Reuters). The selection of studies was conducted by two researchers through screening the title and abstract to exclude irrelevant studies, then the full-text of the remaining studies was systematically evaluated according to the inclusion and exclusion criteria. As shown in Figure 1, a total of 2,111 records were retrieved, and 1767 records were left after duplicates were removed. Then, 1721 records were excluded after screening the title and abstract, leaving 46 articles for full-text review. Among the 46 articles, 31 were excluded due to the following reasons: animal studies (*n* = 6), reviews (*n* = 2), meeting abstracts (*n* = 3), not assessment of muscle mass or muscle strength, and inconsistent intervention methods (n = 6), and 15 articles were used for the systemic review.

### 2.4 Data extraction and quality assessment

The bibliographic information for author, publication year, the characteristics of subjects (sample size, gender, mean age), detailed parameters of WBVT (frequency, amplitude, exposure time and body position or posture during WBVT), and the outcome measurements (muscle mass or muscle cross section area and muscle strength) were independently extracted by two researchers. A summary of the study results was recorded in a standard table format developed for this study. If the information extracted by the two researchers was inconsistent, a third researcher was consulted until it was resolved.

**FIGURE 2:**
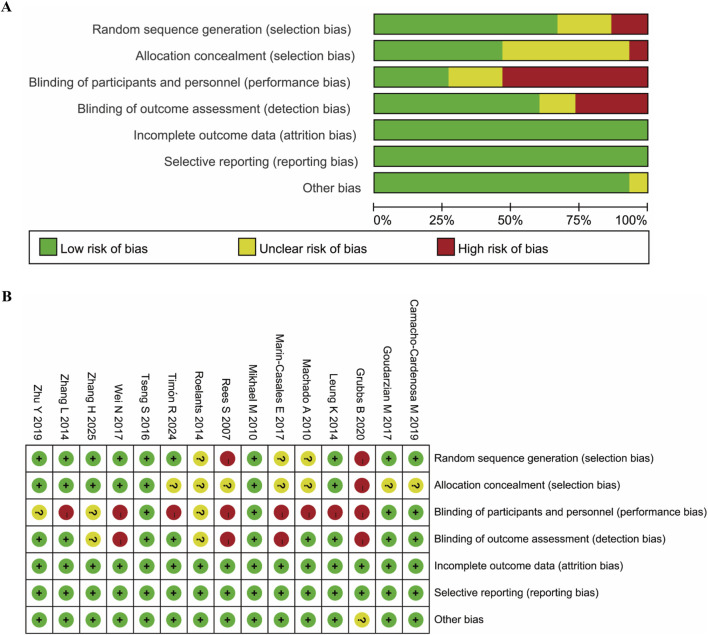
Assessment of risk of bias based on RevMan 5.4. **(A)** Percent of studies with categories for risk of bias; **(B)** Summary for the risk of bias in each study.

The risk of bias was assessed by two researchers independently using the Review Manager (RevMan 5.4; Cochrane, Lindon, United Kingdom) with the following aspects: random sequence generation and allocation concealment (selection bias), blinding of participants and personnel (performance bias), blinding of outcome assessment (detection bias), incomplete outcome data (attrition bias), selective reporting (reporting bias), and other bias. Each study was assigned a bias category of low risk, unclear risk or high risk. The differences in the identification of study biases between the two researchers will be resolved by consulting a third researcher. As shown in Figure 2, the quality of the included studies was found to be acceptable, although 9 studies were shown to be with high risks of performance bias, which is inevitable.

### 2.5 Data synthesis and analysis

All the outcomes are continuous variables and presented as the mean change from baseline in muscle mass and muscle strength. All the data was analyzed using Review Manager (RevMan 5.4; Cochrane, Lindon, United Kingdom), and the standard mean differences (SMD) and 95% confidence intervals (CIs) were used in this review. *P* < 0.05 was considered as statistically significant. The heterogeneity across studies was measured by using the I^2^ statistic, with the value <50% indicating low heterogeneity, 50%–75% implying moderate heterogeneity, and >75% showing fairly high heterogeneity. A fixed-effect model was used when the I^2^ statistic ≤50%, otherwise the random-effect model was used.

### 2.6 Impacts of WBVT on muscle mass and strength in older adults with or without sarcopenia

**FIGURE 3:**
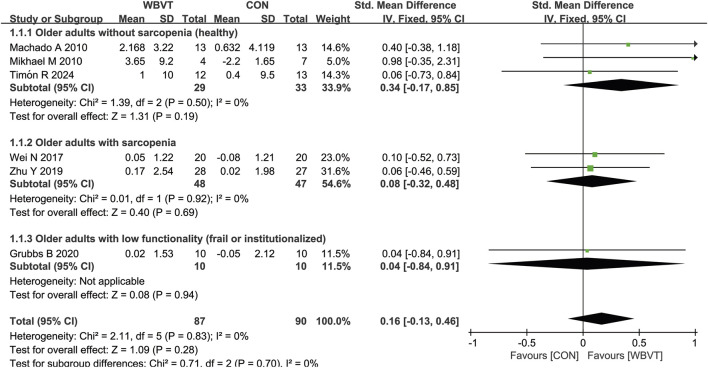
Effects of WBVT on muscle mass in older adults with or without sarcopenia. Values on x-axis denoted standardized mean differences, and the diamond illustrated the 95% confidence interval of the pooled effects.

As shown in Figure 3, three studies measured muscle mass in healthy (non-sarcopenic) older adults, with two studies detected by computed tomography (CT) and one by dual-energy X-ray absorptiometry. Machado et al. (2010) and Timón et al. (2024) demonstrated that there was significant increase in muscle CSA after WBVT. Mikhael et al. reported that WBVT intervention induced potentially clinically meaningful but statistically non-significant improvements in lower leg muscle CSA. Additionally, Bogaerts et al. (2007) also observed a significant increase in thigh muscle mass. However, for older adults with sarcopenia or low functionality, 3 studies (Wei et al., 2017; Zhu et al., 2019; Grubbs et al., 2020), WBVT intervention has no significant impacts on muscle mass, but one study that used skeletal muscle mass index (calculated by total skeletal muscle mass (kg)/body weight (kg) × 100) found that after 12-week WBVT intervention, the muscle mass was significantly higher than that before WBVT intervention (Chang et al., 2018), which may be due to the difference in the form of exercise carried by subjects. The participants in Chang et al.’s study did dynamic exercise on the vibration platform, such as deep squat, wide stance squat and heel raise, whereas in the studies of Wei et al. (2017) and Zhu et al. (2019), subjects were only required to stand in a half-squat standing position during vibrating.

**FIGURE 4:**
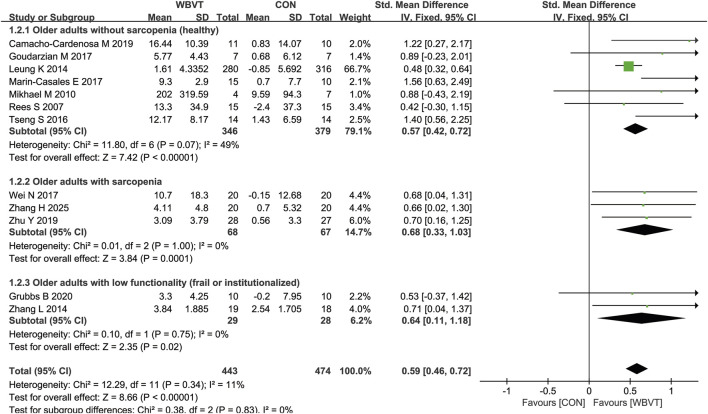
Effects of WBVT on muscle strength in older adults with or without sarcopenia. Values on x-axis denoted standardized mean differences, and the diamond illustrated the 95% confidence interval of the pooled effects.

As shown in Figure 4, a total of 12 studies detected muscle strength in older adults with or without sarcopenia. As shown in Figure 4, all studies demonstrated a significant improvement in healthy (SMD = 0.57, 95% CI − 0.42 to 0.72, I^2^ = 49%, *p* < 0.00001) and sarcopenic (SMD = 0.68, 95% CI−0.33 to 1.03, I^2^ = 0%, *p* < 0.0001) or institutionalized (SMD = 0.64, 95% CI−0.11 to 1.18, I^2^ = 0%, *p* = 0.02) older adults after WBVT intervention compared with CON group.

## 3 The response of muscle to WBVT depends on the detailed parameters

The response of muscle to WBVT not only depends on the detailed parameters, but also varies with body position or posture during WBVT. To clarify the potential effects of moderating variables, we performed subgroup analysis to explore the effects of WBVT on muscle mass and strength in older adults.

**FIGURE 5:**
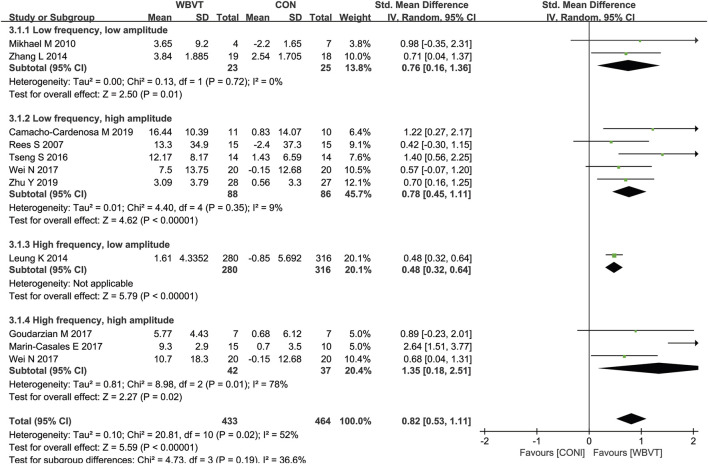
Effects of WBVT with different parameters on muscle strength in older adults. Values on x-axis denoted standardized mean differences, and the diamond illustrated the 95% confidence interval of the pooled effects.

Firstly, the intensity of WBVT is mainly determined by two parameters provided by the vibration machine: frequency and amplitude. The WBVT programmes applied on older adults in most studies were at 12 Hz–60 Hz frequency and with 2–5 mm amplitude. Based on the improvement of muscle power by WBVT (Manimmanakorn et al., 2014), we regarded frequencies less than 30 Hz as low frequency, 30 Hz or higher as high frequency, and regarded amplitude lower than 3 mm as low amplitude, 3 mm or greater as high amplitude. It should be noted that lower frequencies may not transmit the vibration stimuli effectively (Dincher et al., 2020), because during the delivery of vibration stimuli from the lower extremities from bottom to top, the damping of vibration waves through the joints, muscles, and soft tissues leads to a certain degree of attenuation of the stimuli (Oroszi et al., 2020). It has been demonstrated that, contrast to lower vibration frequencies (<30 Hz), higher vibration frequencies (>30 Hz) provided great improvements in muscle strength (Ardigò et al., 2025). As for amplitudes, the higher amplitudes used, the greater activation of lower extremity muscle was induced (Marín et al., 2009), and there was significant increase in lean mass in individuals who experienced high amplitude (4 mm) vibration training, rather than low amplitude (2 mm) (Martínez-Pardo et al., 2013). It was reported that a combination of 60 Hz frequency and 4 mm amplitude of WBVT could induce the greatest myoelectric activity and have the most advantageous effects on muscle strength parameters in young adults (Krol et al., 2011; Stania et al., 2017). Similarly, with the increase of frequency and/or amplitude, WBVT-induced lower limb muscle activity in older adults was enhanced, and the older population showed greater increases in lower limb muscle activity (Lienhard et al., 2017), obtained greater benefits than young counterparts during WBV exercise (Ardigò et al., 2025). As shown in Figure 5, we also found that, compared with WBVT with low frequency low amplitude, low frequency high amplitude and high frequency low amplitude, WBVT with high frequency and high amplitude (≥30 Hz, ≥3 mm) induced the greatest improvement in muscle strength in older adults (SMD = 1.35, 95% CI−0.18 to 2.51, I^2^ = 78%, *p* = 0.02). However, it was demonstrated that when the amplitude was set at 4 mm, the optimal frequency inducing the greatest increase of muscle strength was 40 Hz, but not 60 Hz, which suggested that the vibration frequency and amplitude used for increasing muscle strength in older adults should be set at higher than 30 Hz (lower than 60 Hz) and greater than 3 mm.

Secondly, the choice of body position or posture during WBVT also influences the intensity of muscle contraction. Lam, F.M et al. compared the effects of static erect stand, static deep squat, static semi-squat and single-legged static squat on the activation of lower limb muscles, and showed that static erect stand and single-leg squat induced maximal activation of lower limb muscles (Lam et al., 2016). Roelants, M. et al. also demonstrated that, compared to double leg squats, single-leg squats significantly increased lower limb muscle activity (Roelants et al., 2006). However, for middle-aged and older adults, it is difficult to complete single-leg squats and the quality of the movement is easily compromised, and static upright during WBVT is likely to cause head discomfort in older adults. Liu et al. found that, compared to static semi-squat, dynamic semi-squat significantly increased the muscle activity of gluteus maximus, rectus femoris and vastus medialis (Liu et al., 2022), which suggested dynamic semi-squat may be more beneficial to the development of lower limb muscle group strength in middle-aged women than static semi-squats. In the present review, as shown in Table 1, among 6 of the included studies measuring muscle mass, we found that neither lower frequency, low or high amplitude, nor higher frequency, high amplitude, static posture during WBVT increased muscle mass, however, dynamic posture during WBVT, whether low frequency, high amplitude or progressively increased frequency and amplitude, significantly increased muscle mass, similar as the results reported by Bogaerts et al. that 1-year WBVT with progressive intensity by increasing frequency (35–40 Hz) and amplitude (2.5 or 5 mm) remarkably increased isometric and explosive muscle strength, and muscle mass older men (Bogaerts et al., 2007).

**TABLE 1:** Overview of WBVT parameter within each study of elderly population.

Study	Participants	Posture of WBV	Exercise regimens	Outcomes
Wei et al. (2017)	Older people with sarcopenia	Stood barefoot with their knee joint flexed at 60° on the platform	20, 40, 60 Hz, 4 mm, 3 days/week, 12 weeks	Isometric knee extension ↑ Muscle CSA —
Zhu et al. (2019)	Sarcopenic men	Stand on the vibration plate with legs and knees slightly bent	12–16 Hz, 3–5 mm, 5 days/week, 8 weeks	Muscle mass — Muscle strength ↑
Zhang et al. (2025)	Elderly people with sarcopenia	Stand on the vibration plate with the knee joints flexed 45°	9–14 Hz, 2–3 mm, 5 days/week, 8 weeks	Muscle strength ↑
Rees et al. (2007)	Healthy older adults	Stand on the vibration plate and perform static squats and dynamic squatting and calf raises	26 Hz, 5–8 mm, 3 times/week, 8 weeks	Isokinetic muscle strength ↑
Leung et al. (2014)	Postmenopausal females over 60 years	Stand on the vibration platform without knee bending	35 Hz, 0.3 g, 5 days/week, 18 months	Muscle strength ↑
Camacho-Cardenosa et al. (2019)	Healthy elderly	Stand with barefoot and knee flexion of 120°	12.6 Hz, 4 mm, twice a week, 18 weeks	Maximal strength of knee extensors ↑
Goudarzian et al. (2017)	Elderly women	(1) Standing straight with knees semi-locked; (2) isometric squat at a knee angle of approximately 120°; (3) kneeling on the floor with arms straight and hands placed on the platform; (4) squatting at a rhythm of 2 s up and 2 s down at a knee angle of approximately 120°; (5) lunge position with the left leg on the platform and the right leg on the ground; and (6)lunge position with the right leg on the platform and the left leg on the ground	30–35 Hz, 5 mm, 10 days	Muscle strength ↑
Tseng (2016)	Healthy older adults	Stand on the vibration platform with the knees slightly flexed (about 20°)	20 Hz, 4 mm, 3 times/week, 12 weeks	knee extensors strength ↑
Marín-Cascales et al. (2017)	Postmenopausal women	Stood on the platform holding a half-squat (knee and hip angle 120°), the arms remained crossed and parallel to the floor with a shoulder flexion of 90°	35–40 Hz, 4 mm 3 times/week, 24 weeks	Muscle strength ↑
Roelants et al. (2004)	Postmenopausal women	Stand on the vibration platform and performed a total-body-training program consisting of unloaded static and dynamic exercises	35 Hz–40 Hz, 2.5–5.0 mm, 3 times/week, 24 weeks	Isometric and dynamic knee extensor strength ↑
Machado et al. (2010)	Community-dwelling elderly women older than 65 years	Stand on the vibration platform and performed a lower-body-training program consisting of unloaded static and dynamic exercises including a half-squat (knee angle between 120° and 130°) and a deep squat (knee angle 90°), a wide-stance squat, and calves	20–40 Hz, 2–4 mm, 3–5 times/week, 10 weeks	Muscle strength ↑ muscle CSA ↑
Mikhael et al. (2010)	Non-institutionalized adults aged 50 and older	Stand on the vibration platform with the knees locked or slightly flexed 20°	12 Hz, 1 mm, 3 times per week, 13 weeks	Relative upper body strength ↑ Absolute lower body strength ↑ Lower leg muscle cross-sectional area —
Timón et al. (2024)	Elderly adults older than 65 years	Stood on the platform and performed 4 different exercise: (1) static squatting position at 120°, upright trunk, and simultaneously performing biceps curl with elastic band attached to the feet; (2) lunge with one leg positioned forward with knee bent 90°and foot flat on the vibration platform while the other leg is positioned behind on the floor, simultaneously performing standing row with elastic band (12–15 reps). (3) glute bridge exercise with feet on the vibration platform and the upper back resting on the floor, creating a straight line from knees to shoulders; (4) front plank, with feet on the vibration platform and forearms resting on the floor	18–22 Hz, 4 mm, 3 times/week, 20 weeks	Muscle mass ↑
Zhang et al. (2014)	Frail elderly patients	Stand on the platform with or without flexion at the hips, knees and ankle joints	6–26 Hz, 1–3 mm, 3–5 times/week, 8 weeks	Knees extensor strength ↑
Grubbs et al. (2020)	Frail elderly adults	Stand on the platform and perform 4 lower body exercises (partial squat, narrow squat, wide squat, calf raise)	25–40 Hz, 12 weeks	isometric knee extension strength ↑ Skeletal muscle index —

Finally, exposure time is also an important factor influencing the effects of WBVT. Too short exposure may not elicit any change in muscle performance, but too long exposure time induced muscle fatigue. Silva-Grigoletto et al. reported that muscle performance detected by jump height and power output in half squat (90° knee flexion) was improved after 30 s and 60 s WBVT, whereas decreased after experiencing 90 s vibration stimulus, and 6 sets of WBVT showed a greater effect on jump ability and power output (Stania et al., 2017). Stewart et al. showed that WBVT (at 26 Hz with peak-to-peak amplitude of 4 mm) produced an improvement in isometric right knee extension strength only after the 2 min, but decreased with 4 and 6 min (Stewart et al., 2009), the inconsistence of the exposure time may be associated with the difference in the platform used and the outcomes assessed in the two studies. For older adults with sarcopenia, it was indicated that 12-week WBVT with a combination of 40 Hz frequency, 4 mm amplitude and 360 s exposure time significantly induced voluntary activation of quadriceps muscles, and improved muscle strength and mass (Wei et al., 2017; Wei and Ng, 2018).

Although it has been well-demonstrated that WBVT with different parameters has beneficial effects on improving muscle mass and strength in individuals with sarcopenia and with high safety, it was not widely applied in clinic, which may be because there is no standard training programme established at present, as the outcome of WBVT on muscle mass and strength determined by multiple factors, not only depends on the detailed parameters of WBVT, such as the vibration frequency, amplitude, exposure time, but also varies with body position or posture during WBVT. In addition, the beneficial effects of WBVT usually required 2–3 months period or even longer, the limited hospital beds limited its application in clinic. To solve this problem, researchers or rehabilitation specialists should firstly establish the unified and standardized training programme by conducting large-scale studies. Then the government or hospitals could place vibration equipment in the community, and older adults with sarcopenia could use it to perform WBVT after importing personal information that linked with the medical system of hospitals.

## 4 The possible mechanisms of whole-body vibration training on improvements of muscle mass and strength in older individuals

### 4.1 Neuromuscular junction transmission

Skeletal muscle is a type of muscle tissue whose activities is controlled by nervus. The neuromuscular junction (NMJ), as the nexus between the nervous and muscular systems responsible for converting nerve action potentials from the presynaptic motor neuron to trigger contraction of the postsynaptic muscle fiber, is critical for the input and dependable neural control of muscle force generation. Most evidence from animal models demonstrated that the NMJ undergoes profound morphological changes with advancing age, such as the increased dimensions and complexity of nerve terminal branching, the decreased number of acetylcholine (ACh)-containing vesicles, ACh receptors and junctional folds, NMJ denervation and increased endplate fragmentation. Furthermore, ageing triggers adaptations to NMJ physiological function, leading to a decreased safety factor and an increased incidence of neurotransmission failure (Pratt et al., 2021). Despite NMJ aging is well demonstrated in rodents, much less is known about the process in humans, due to the absence of muscle needle biopsies. Earlier studies comparing autopsy samples of young and old humans showed that aged NMJ exhibited increased pre-synaptic branching, peri-junctional AChRs and endplate disruption (Arizono et al., 1984; Wokke et al., 1990). Sarto et al. investigated the neuromuscular system integrity and function, especially alterations in NMJ stability and MU properties at different stages of sarcopenia in human, and indicated that neuromuscular alterations (MU loss, NMJ instability, impaired NMJ transmission, myofibre denervation and axonal damage) are present in non-sarcopenic, pre-sarcopenic and sarcopenic individuals aged >70 years without major comorbidities, and these neuromuscular alterations are accompanied by muscle wasting and weakness (Arnold and Clark, 2023; Sarto et al., 2024), therefore improving neuromuscular maladaptations may be an avenue to prevent the onset and progression of sarcopenia. It was demonstrated that increasing motor neuron viability and the restoration of neuromuscular junctions and function enhanced the muscle strength of aged mice (Bakooshli et al., 2023).

Exercise could be an effective approach to combat age-related muscle weakness by preventing both impairments of neuromuscular transmission and alterations in myofiber composition. Yamaguchi et al. demonstrated that 8-week low-intensity treadmill running counteracted the age-related decline in grip strength and myofibre composition shifts, which was associated with reversing the alterations in NMJ morphology, increasing the degree of synaptic overlap and decreasing the percentage of denervated myofibres (Yamaguchi et al., 2025). Similarly, voluntary wheel running was also demonstrated to improve NMJ transmission although does not prevent motor unit losses in aged mice (Chugh et al., 2021), and preserved innervation at NMJ and muscle contractile function following ischemia-reperfusion injury (Wilson et al., 2019). WBVT was also shown to improve neuromuscular transmission, and widely applied in athletes to enhance sports performance (Oliveira et al., 2022), in patients with Parkinson’s disease (Chang et al., 2022), stroke (Wei and Cai, 2022; Zhang M. et al., 2022) and multiple sclerosis (Andreu et al., 2020) for rehabilitation or remedy. Evidence from animal experiments demonstrated that WBVT at 60 Hz frequency with 2 mm amplitude increased the size of the area of section and the mean diameter of NMJ in control and obese Wistar rats, but didn’t alter the concentration of the cholinesterase enzyme in the synaptic cleft (Boaretto et al., 2020). Peretti et al. found that 4-week WBVT at a frequency of 60 Hz and duration of 10 min could reverse oophorectomy-induced decrease of the area and diameter of NMJs is Wistar rats (Peretti et al., 2019). Furthermore, surface electromyography (EMG) is a useful tool for the evaluation of the neuromuscular activation of the muscle fibers, mounting evidence demonstrated that WBVT could improve neuromuscular transmission determined by EMG activity in young (Krol et al., 2011) and older adults (Monteiro-Oliveira et al., 2022; Liu et al., 2023). The neuromuscular activation during WBV was shown to be closely related to the frequency and amplitude of vibration, the higher the frequency, the higher the EMG activity (Krol et al., 2011; Ritzmann et al., 2013). The optimal combination of the frequency and amplitude of WBVT inducing myoelectric activity was 60 Hz and 4 mm for female students, respectively (Krol et al., 2011). The body position or posture during WBVT was also related to the muscle activation. Compare with 10° and 30° of knee flexion, the knee joint with 60° flexion elicited the highest neuromuscular activity (Ritzmann et al., 2013), dynamic semi squat increased the activation of lower muscle in older people, compared to static semi squat WBVT (Liu et al., 2023). Therefore, when designing optimal WBVT protocol for elderly subjects, the detailed parameters and age should be taken into consideration.

### 4.2 Muscle fiber capillarization

Skeletal muscle capillarization plays a key role in oxygen and nutrient delivery to muscle. The capillary rarefaction limits the transcapillary transport of nutrients and oxygen to muscle and may contribute to sarcopenia and functional impairment in older adults. Mounting evidence indicated that capillary density in skeletal muscles and microvascular function are comprised with aging (Groen et al., 2014; Fukada and Kajiya, 2020), and this age-related capillary rarefaction was associated with muscle atrophy (Fukada and Kajiya, 2020), lower whole-body lean tissue mass, appendicular lean tissue mass and appendicular lean tissue mass divided by body mass index in healthy older men (Betz et al., 2021). Prior et al. demonstrated that sarcopenic subjects had 20% lower capillary-to-fiber ratio, as well as 13% and 15% lower VO2max expressed as mL/kg/min or L/min, respectively, compared with sex-, race-, and age-matched participants without sarcopenia, and lower thigh muscle cross-sectional area and VO2max correlated directly with reduced capillarization (Prior et al., 2016), which suggested low skeletal muscle capillarization is one factor that may contribute to sarcopenia and reduced exercise capacity in older adults. Impaired muscle performance was restored by angiogenic treatments alleviating microvascular rarefaction (Tickle et al., 2020), thus muscle capillaries might be a therapeutic target to counteract the decline of skeletal muscles with age.

WBVT could also improve intramuscular blood perfusion and was widely applied for rehabilitation in patients with chronic stroke (Huang et al., 2020), spinal cord injury and Friedreichs ataxia (Herrero et al., 2010). A single session of WBV is sufficient to significantly enhance muscle microvascular blood flow in healthy individuals (Betik et al., 2021), and the effects of WBVT on muscle blood flow depend on vibration type and frequencies. A meta-analysis revealed that a side-alternating WBV may cause a different or greater response than vertical vibrations (Games et al., 2015), but the mechanisms how side-alternating vibration elicits different physiologic responses than vertical vibration. WBVT with lower frequencies (5–25 Hz) produces a greater effect on peripheral blood flow than higher frequencies (30–50 Hz), which may be associated with the rate of muscle contraction. WBVT with lower frequencies provides more time between contractions and allows for greater perfusion, while higher frequencies may not allow for this perfusion and result in lower blood flow during WBV application (Games et al., 2015). Lythgo et al. also demonstrated that WBVT with lower frequencies (10–30 Hz) increased blood velocity in the femoral artery more than did higher vibration frequencies (20–30 Hz) (33% versus 27%) (Lythgo et al., 2008). Additionally, the angiogenesis is regulated by a balance between pro- and anti-angiogenic factors. The limited evidence showed that the preventive effect on capillary reduction by whole-body vibration was probably through decreasing anti-angiogenic factor CD36 and increasing pro-angiogenic factor vascular endothelial growth factor-A (VEGF-A) (Kaneguchi et al., 2014).

### 4.3 Increasing protein synthesis and decreasing protein degradation

The protein synthesis and degradation are important factors affecting muscle mass during aging. The increase of protein synthesis and the decrease of protein degradation induced muscle mass gain, on the contrary, it resulted in muscle loss and the decline of muscle strength (Han J.-W. et al., 2024; Song et al., 2024; Yang et al., 2024). Low protein intake is associated with a higher risk of sarcopenia and low hand grip strength in older adults (Han M. et al., 2024), and protein supplementation is recommended to attenuate muscle loss during aging. However, protein supplementation alone does not improve affect appendicular muscle mass, handgrip strength, and physical performance in the elderly with sarcopenia (Kamińska et al., 2023), which may be associated with diminished response to anabolic stimulus in older subjects. Exercise, particularly resistance training remains a cornerstone intervention. Growing evidence indicates that exercise alone or exercise combined with protein supplementation shows promising effects on muscle mass and strength in older adults with sarcopenia (Whaikid and Piaseu, 2024; Yoshimura et al., 2025). Mammalian target of rapamycin (mTOR) is the primary positive regulator of protein synthesis, and forkhead box O1 (FoxO1) is an important regulator of protein degradation in skeletal muscle through regulating muscle-specific ubiquitin E3 ligases: muscle atrophy F-box (MAFbx) or muscle ring-finger protein 1 (MuRF1). Insulin-like growth factor 1 (IGF-1)-mediated phosphoinositide 3-kinase PI3K (PI3K)/protein kinase B (PKB/Akt) is the common signaling pathway regulating the activities of mTOR and FoxO1, and plays crucial roles in muscle mass and strength (Zhang H. et al., 2022; Hwangbo et al., 2023). Most evidences indicated that the muscle hypertrophy induced by exercise was fulfilled through regulating IGF-1/PI3K/Akt/mTOR (Yin et al., 2020) or IGF-I/Akt/FoxO pathway (Biglari et al., 2020).

There is evidence demonstrated that, similar to conventional resistance and aerobic exercise, WBVT increased skeletal muscle mass, exercise capacity and protein synthesis, and inhibited protein degradation in mice undergoing early aging via activating IGF-1/IGF-1R–PI3K/Akt pathway (Li et al., 2022). Results from *in vivo* and *in vitro* experiments also showed that low-amplitude high frequency vibration strongly downregulates the transcript levels of atrophy gene myostatin and atrogin-1 (Ceccarelli et al., 2014). Our previous study also indicated that WBVT promoted the increase of muscle mass and strength in female ovariectomized mice through activating Akt-mTOR and suppressing FoxO1 signal pathways, but the improving effect of WBVT on muscle mass and strength was affected by estrogen status, because the activated degree of Akt-mTOR and the inhibited degree of FoxO1 in ovariectomized mice with estrodiol supplementation were greater than that without estrogen administration (Tian et al., 2024). However, Ende et al. demonstrated that WBVT had minor effects on grip strength in C26 tumor bearing mice, although prevented the upregulation of the proteasome pathway in the SOL, which may be resulted from the shorter duration time of WBVT, because WBVT performed in C26 cachectic mice only lasted for 19 days (van der Ende et al., 2021), but it should be verified in the further studies.

### 4.4 Promoting myogenesis

Skeletal muscle has a remarkable capacity to regenerate after injuries, which is mediated by the resident muscle stem cells, that are also called satellite cells. Satellite cells, located between the sarcolemma and the basal lamina of the muscle fiber, are usually in a quiescent state, and become activated in response to mechanical strain, then differentiating into myocytes and fusing with each other to form new myotubes or with existing myofibers to add new nuclei, thus promoting muscle hypertrophy and muscle gain. The reduced number of satellite cells and the impaired regenerative capacity are attributed to important factors causing age-related muscle atrophy or sarcopenia (Huo et al., 2022), and satellite cell transplantation or boosting muscle regeneration are considered as the two important strategies to treat age-related muscle loss or sarcopenia (Hall et al., 2010; Naranjo et al., 2017).

Satellite cells are sensitive to mechanical stresses including strain, fluid flow, pulse, and vibration, and convert these stimuli into biochemical signals that affect cellular morphology, proliferation and differentiation. Mounting evidence demonstrated that various types of mechanical stimulations, such as stretching and fluid shear stress, effectively promote satellite cell proliferation and differentiation, and muscle regeneration (Fu et al., 2021; Fu et al., 2024). Recently, mounting evidence indicated that WBVT could regulate myoblast proliferation and differentiation, and promotes muscle repair and regeneration. WBVT at a selected frequency (30 or 50 Hz) with low (approximately 2.5 mm) or high (approximately 5.0 mm) amplitude promotes the proliferation and migration of satellite cells and accelerate the process of muscle regeneration in cardiotoxin-induced mice, results from *in vitro* experiments also showed that vibration stimulation at 30 Hz with 2.5 mm amplitude promoted the proliferation of C2C12 myoblasts in a time-dependent manner (Sato et al., 2024). Similarly, Ceccarelli et al. also demonstrated vibration stimulation at 30 Hz effectively enhanced the fusion of satellite cells (Ceccarelli et al., 2014).

Myogenesis is a highly coordinated and intricate process that regulated by several myogenic regulatory factors (MRFs), such as myogenic differentiation (MyoD), myogenic factor 5 (Myf5) and myogenin. MyoD and Myf5 commit cells to the myogenic program, while myogenin controls the differentiation and fusion of myoblasts to form myofibers. Enhancing the expressions of MyoD and myogenin facilitated myoblast differentiation (Kurosaka et al., 2023), whereas downregulating their expressions inhibited myogenic differentiation and reduced myotube number and area (Luo et al., 2019). Satellite cells reside in a specialized niche that includes different components, cells and surrounding extracellular matrix (ECM), which direct satellite cell behavior through reciprocally interacting with satellite cells. The ECM has been shown to be capable of adapting to changes in the external environment, such as mechanical loading or inactivity and disuse, specifically with collagen levels responding to altered levels of physical activity. The regulatory role of collagen in muscle differentiation depends on the interaction between type I collagen and proteoglycans decorin. It has been reported that decorin administration can influence the rate and extent of collagen fibrillogenesis (Zhang et al., 2009), and promote the proliferation and differentiation of myoblasts through suppressing myostatin, a member of the TGF-superfamily, that has inhibitory effects on myoblast proliferation and differentiation (Kishioka et al., 2008). Vibration stimulus at frequencies of 8–10 Hz stimulated the expression of MRFs and the ECM protein type I collagen and decorin, increasing myotube formation (Wang et al., 2010). PI3K/Akt, except regulating the protein synthesis and degradation, also plays important roles in myoblast proliferation and differentiation. Activating PI3K/Akt promotes the proliferation and differentiation of satellite cells (Wang et al., 2024), whereas inhibiting PI3K/Akt activity suppresses their proliferation and differentiation (Wei et al., 2023). Lin et al. demonstrated that vertical vibration at 10 Hz with 0.4 mm amplitude for 10 min/day promotes the activation of PI3K/Akt signal pathway, and increased the protein levels of ECM protein type I collagen and decorin, MRFs MyoD and myogenin, accompanied with the increase of myotube number, whereas administration with PI3K/Akt inhibitor LY294002 inhibited vibration stimulus-induced increase of these indicator, indicating vertical vibration-induced myotube formation may be mediated through PI3K/Akt signal pathway (Lin et al., 2021). Additionally, stathmin, a highly conserved protein in various species and involving in regulating cell cycle, microtubules and apoptosis, was reported to be associated with vertical vibration-initiated myogenesis, because interfered with stathmin siRNA remarkably reduced vibration stimulus-induced myotube formation (Lin et al., 2021).

## 5 Conclusion and prospective

**FIGURE 6:**
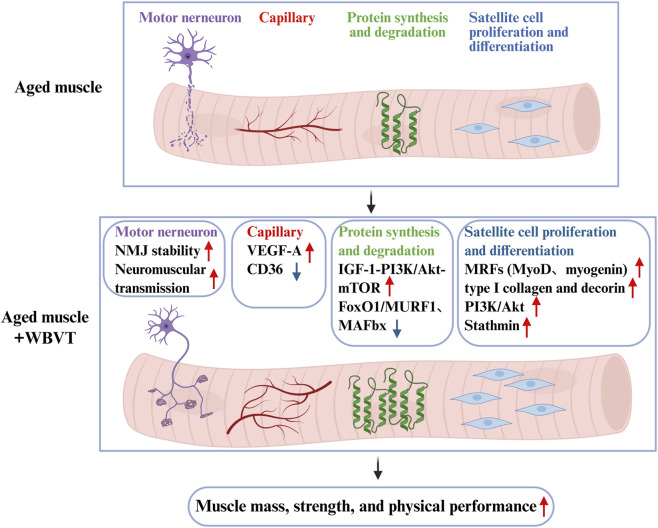
The scheme of the underlying mechanisms about WBVT-induced improvements on muscle mass and strength in elderly people. WBVT might improve muscle mass and strength in elderly people through intra- (promoting myogenesis by increasing the levels of MRFs, stathmin, type I collagen and decorin, and activating PI3K/Akt pathway, increasing protein synthesis (via activating PI3K/Akt/mTOR pathway) and decreasing protein degradation (by FoxO1-mediated MURF1 and MAFbx expression)) and extra-muscular pathways (improving muscle capillary via downregulating anti-angiogenic factor CD36 and upregulating pro-angiogenic factor (VEGF-A), and enhancing neuromuscular transmission via improving NMJ structure).

As global ageing accelerates, the prevalence of sarcopenia raises parallelly, searching for effective approaches to delay or prevent sarcopenia in older adults is extremely urgent. WBVT was proposed as a promising treatment strategy for sarcopenic individuals. All the included studies in this review demonstrated that WBVT could effectively improve muscle strength in older adults with or without sarcopenia, and the improvement of WBVT on muscle strength was greatest when set at high frequency with high amplitude (≥30 Hz, ≥3 mm). However, WBVT only has beneficial effects on muscle mass only in older adults without sarcopenia, but not sarcopenic older adults, which may be associated with the body position during WBVT. Compared with static body position, dynamic body position during WBVT has promoting effect on muscle mass in older adults, even at low frequency. As shown in Figure 6, the possible mechanisms of WBVT on muscle mass and strength in older people involves (1) enhancing NMJ stability, (2) improving muscle fiber capillarization by decreasing anti-angiogenic factor CD36 and increasing pro-angiogenic factor VEGF-A, (3) increasing protein synthesis via IGF-1/PI3K/Akt/mTOR pathway and decreasing protein degradation through PI3K/Akt/FoxO1 pathway, (4) promoting myogenesis through activating PI3K/Akt, upregulating ECM protein type I collagen and decorin, and increasing the level of stathmin.

In fact, the pathogenesis of sarcopenia is multifactorial, involving in malnutrition, hormonal changes, mitochondrial dysfunction, inflammation infiltration and alterations in intestinal microbiota. Among them, mitochondria, as the cellular power factory, not only dedicates to ATP production, but also is the major site of reactive oxidative species (ROS) generation. Under physical conditions, the ROS could be effectively scavenged by antioxidative system in our body, such as superoxide dismutase (SOD), catalase (CAT), glutathione peroxidase (GSH-Px), Peroxiredoxin family 1–6 (Prdx 1–6) and Sestrin 1–3. However, mitochondrial function is impaired during aging. Dysfunctional mitochondrial produced less ATP and releases excessive ROS simultaneously, which are major contributing factors for sarcopenia. In addition, age-related decline of estrogen or testosterone is closely associated with sarcopenia. Despite estrogen or testosterone replacement therapy could improve muscle mass and strength in individuals with sarcopenia, it brings several adverse effects. Mounting evidence demonstrated that appropriate aerobic or resistance exercise could elevate the level of serum testosterone or estradiol and promoted muscle hypertrophy, but the effects of WBVT on muscle mass and strength was associated with improving mitochondrial function, alleviating oxidative stress and increasing serum estradiol or testosterone remained unclear, and should be clarified in the future studies.
