# Supplementary material for: Whole-body vibration training improves muscle mass and strength in older adults through intra- and extra-muscular pathways
Source: Front Cell Dev Biol. 2025 Oct 24;13:1643478. doi: 10.3389/fcell.2025.1643478 (PMC12591996; doi:10.3389/fcell.2025.1643478)
Supplement: Supplementary file 1 [file Supplementaryfile1.docx]

**Pubmed**

(((((((((((whole-body vibration intervention) OR (vibration)) OR (vibration therapy)) OR (vibrating)) OR (whole body vibration)) OR (whole-body vibration)) OR (whole-body vibration training)) OR (vibration training)) OR (whole-body vibrating therapy)) AND (((((((old adults) OR (older people)) OR (older adults)) OR (elderly people)) OR (elderly)) OR (aging adults)) OR (advanced old age))) AND ((((((((((((muscle mass) ) OR (muscle size)) OR (muscle loss)) OR (muscle wasting)) OR (muscular atrophy)) OR (muscle weakness)) OR (sarcopenia)) OR (sarcopenic)) OR (muscle strength)) OR (strength)) OR (handgrip strength))) NOT ((((((((((((Chronic Obstructive Pulmonary Diseases) OR (COPD)) OR (Tumor)) OR (Cancer)) OR (Malignancy)) OR (Kidney Diseases)) OR (kidney disease)) OR (stroke)) OR (stroke patients)) OR (cerebral palsy)) OR (multiple sclerosis)

**Web of science**

#1 whole-body vibration intervention (Topic) or vibration (Topic) or vibration therapy (Topic) or vibrating (Topic) or whole body vibration (Topic) or whole-body vibration (Topic) or whole-body vibration training (Topic) or vibration training (Topic) or whole-body vibrating therapy (Topic)

#2 old adults (Topic) or older people (Topic) or older adults (Topic) or elderly people (Topic) or elderly (Topic) or aging adults (Topic) or advanced old age (Topic)

#3 muscle mass (Topic) or muscle size (Topic) or muscle loss (Topic) or muscle wasting (Topic) or muscular atrophy (Topic) or muscle weakness (Topic) or sarcopenia (Topic) or sarcopenic (Topic) or muscle strength (Topic) or strength (Topic) or handgrip strength (Topic) or muscle function (Topic) or muscle performance (Topic)

#4 Chronic Obstructive Pulmonary Diseases (Topic) or COPD (Topic) or Tumor (Topic) or Cancer (Topic) or Malignancy (Topic) or Kidney Diseases (Topic) or kidney disease (Topic) or stroke (Topic) or stroke patients (Topic) or chronic stroke (Topic) or cerebral palsy (Topic) or multiple sclerosis (Topic) or diabetes (Topic)

#1 AND #2 AND #3 NOT # 4

**Embase**

#1 'muscle mass'/exp OR 'muscle mass' OR (('muscle'/exp OR muscle) AND ('mass'/exp OR mass)) OR 'muscle size':ab,ti OR 'muscle loss':ab,ti OR 'muscle wasting':ab,ti OR 'muscular atrophy':ab,ti OR 'muscle weakness':ab,ti OR sarcopenia:ab,ti OR sarcopenic:ab,ti OR 'muscle strength':ab,ti OR strength:ab,ti OR 'handgrip strength':ab,ti OR 'grip strength':ab,ti OR 'muscle function':ab,ti OR 'muscle performance':ab,ti

#2 ('whole-body vibration intervention' OR ('whole body' AND ('vibration'/exp OR vibration) AND ('intervention'/exp OR intervention)) OR vibration:ab,ti OR 'vibration therapy':ab,ti OR vibrating:ab,ti OR 'whole body vibration':ab,ti OR 'whole-body vibration':ab,ti OR 'whole-body vibration training':ab,ti OR 'vibration training':ab,ti OR 'whole-body vibrating therapy':ab,ti) NOT 'chronic obstructive pulmonary diseases':ab,ti NOT copd:ab,ti NOT tumor:ab,ti NOT cancer:ab,ti NOT malignancy:ab,ti NOT 'kidney diseases':ab,ti NOT 'kidney disease':ab,ti NOT stroke:ab,ti NOT 'chronic stroke':ab,ti NOT 'cerebral palsy':ab,ti NOT 'multiple sclerosis':ab,ti NOT diabetes:ab,ti

#3 'old adults' OR (old AND ('adults'/exp OR adults)) OR 'older people':ab,ti OR 'older adults':ab,ti OR 'elderly people':ab,ti OR elderly:ab,ti OR 'aging adults':ab,ti OR 'advanced old age':ab,ti OR aging:ab,ti OR aged:ab,ti

#1 AND #2 AND #3
